# Intermittent Fasting Protects against Alzheimer’s Disease Possible through Restoring Aquaporin-4 Polarity

**DOI:** 10.3389/fnmol.2017.00395

**Published:** 2017-11-29

**Authors:** Jingzhu Zhang, Zhipeng Zhan, Xinhui Li, Aiping Xing, Congmin Jiang, Yanqiu Chen, Wanying Shi, Li An

**Affiliations:** ^1^Department of Nutrition and Food Hygiene, School of Public Health, China Medical University, Shenyang, China; ^2^Department of Nutrition and Food Hygiene, School of Public Health, Jinzhou Medical University, Jinzhou, China; ^3^Department of Clinical Nutrition, First Affiliated Hospital, China Medical University, Shenyang, China

**Keywords:** Alzheimer’s disease, intermittent fasting, β-hydroxybutyrate, aquaporin-4, microRNA-130a, histone deacetylase 3

## Abstract

The impairment of amyloid-β (Aβ) clearance in the brain plays a causative role in Alzheimer’s disease (AD). Polarity distribution of aquaporin-4 (AQP4) is important to remove Aβ from brain. AQP4 polarity can be influenced by the ratio of two AQP4 isoforms M1 and M23 (AQP4-M1/M23), however, it is unknown whether the ratio of AQP4-M1/M23 changes in AD. Histone deacetylase 3 has been reported to be significantly increased in AD brain. Moreover, evidence indicated that microRNA-130a (miR-130a) possibly mediates the regulation of histone deacetylase 3 on AQP4-M1/M23 ratio by repressing the transcriptional activity of AQP4-M1 in AD. This study aimed to investigate whether intermittent fasting (IF), increasing the level of an endogenous histone deacetylases inhibitor β-hydroxybutyrate, restores AQP4 polarity via miR-130a mediated reduction of AQP4-M1/M23 ratio in protection against AD. The results showed that IF ameliorated cognitive dysfunction, prevented brain from Aβ deposition, and restored the AQP4 polarity in a mouse model of AD (APP/PS1 double-transgenic mice). Additionally, IF down-regulated the expression of AQP4-M1 and histone deacetylase 3, reduced AQP4-M1/M23 ratio, and increased miR-130a expression in the cerebral cortex of APP/PS1 mice. *In vitro*, β-hydroxybutyrate was found to down-regulate the expression of AQP4-M1 and histone deacetylase 3, reduce AQP4-M1/M23 ratio, and increase AQP4-M23 and miR-130a expression in 2 μM Aβ-treated U251 cells. Interestingly, on the contrary to the result observed in 2 μM Aβ-treated cells, AQP4 expression was obviously decreased in cells exposed to 10 μM Aβ. miR-130a mimic decreased the expression of AQP4-M1 and the ratio of AQP4-M1/M23, as well as silencing histone deacetylase 3 caused the up-regulation of AQP4 and miR-130a, and the reduction of AQP4-M1/M23 ratio in U251 cells. In conclusion, IF exhibits beneficial effects against AD. The mechanism may be associated with recovery of AQP4 polarity, resulting from the reduction of AQP4-M1/M23 ratio. Furthermore, β-hydroxybutyrate may partly mediate the effect of IF on the reduction of AQP4-M1/M23 ratio in AD, in which miR-130a and histone deacetylase 3 may be implicated.

## Introduction

Alzheimer’s disease (AD) is a common neurodegenerative disorder in the elderly and characterized by progressive cognitive dysfunction and behavioral impairment. AD comprises early-onset AD (familial AD) which affects a minority of AD patients and late-onset AD (sporadic AD) which afflicts over 95% of patients with AD (Bruni et al., 2014; Guerreiro and Hardy, 2014). Excessive accumulation of amyloid-β (Aβ) in brain is found in both early-onset AD and late-onset AD, which has been assumed to result from an imbalance between its production and clearance (Zlokovic et al., 2000). Increasing evidence has supported that Aβ production and clearance are impaired in early-onset AD, whereas individuals with late-onset AD exhibit impaired Aβ clearance only (Mawuenyega et al., 2010; Potter et al., 2013). Therefore, Aβ clearance has been considered to be crucial in the development of AD, and targets enhancing Aβ clearance may be alternative strategies for AD treatment.

So far, several mechanisms have been identified as being involved in clearing Aβ from the brain, including enzymatic degradation, cellular uptake, transport across the blood–brain barrier and blood–cerebrospinal fluid barrier, interstitial fluid bulk flow, and cerebrospinal fluid absorption into the circulatory and lymphatic systems (Tarasoff-Conway et al., 2015). Animal studies found that about 75% of extracellular Aβ is cleared by the blood–brain barrier, with only about 10% being cleared by interstitial fluid bulk flow (Shibata et al., 2000; Zlokovic and Frangione, 2003). However, recent studies have indicated that interstitial fluid bulk flow contributes to a larger portion of extracellular Aβ clearance than previously thought (Iliff et al., 2012; Kress et al., 2014). Aquaporin-4 (AQP4)-dependent glymphatic (glial + lymphatic) pathway has been demonstrated to play an important role in driving the removal of soluble Aβ from the interstitium (Rodriguez et al., 2006; Iliff and Nedergaard, 2013). AQP4 is the most highly expressed aquaporin in the mammalian brain, with the polarized distribution in the perivascular endfeet of astrocytes (Moftakhar et al., 2010). More importantly, Aβ clearance was reduced by 55–65% in AQP4 knockout mice (Iliff et al., 2012). In AD, although the alteration of AQP4 expression in the brain remains controversial, several lines of evidence has shown that AQP4 mislocalization performs a crucial role in the reduction of Aβ clearance (Yang et al., 2011; Xu et al., 2015).

AQP4 is expressed as two major principal isoforms: a long isoform with translation initiation at Met-1 (AQP4-M1) and a short isoform with translation initiation at Met-23 (AQP4-M23), forming heterotetramers in the plasma membrane. AQP4 tetramers further aggregate into supra-molecular structures named orthogonal arrays of particles (OAPs) (Nicchia et al., 2010; Yang et al., 2012). Papadopoulos and Verkman (2013) found that AQP4-M23 is enriched in the core of OAPs, and AQP4-M1 in the periphery. In addition, increased proportion of AQP4-M1/M23 in oocytes led to disorganize OAPs (Furman et al., 2003). It has been documented that OAPs plays a role in the AQP4 polarity to astrocyte endfeet (Frydenlund et al., 2006; Noell et al., 2009). Furthermore, AQP4-M1/M23 ratio was found to be elevated with the increase of total AQP4 protein expression after hypoglycemia which may cause disorganization of the OAPs and loss of AQP4 polarity (Deng et al., 2014). However, it is still not clear whether the ratio of AQP4-M1/M23 expression is increased in AD.

Some studies have identified microRNA recognition sites in gene promoters proximal to known transcription initiation sites and implicated microRNAs can regulate the transcription of the target gene (Kim et al., 2008; Younger and Corey, 2011). The transcriptional activity of AQP4-M1 was found to be repressed by microRNA-130a (miR-130a), which may result in the reduction of AQP4-M1/M23 ratio, in Astrocytoma (CRL1718) and HeLa (CCL2) cells (Sepramaniam et al., 2012). As we know, the expression of microRNAs can be regulated by histone acetylation. Meanwhile, the level of histone acetylation is significantly reduced in the brain of AD (Zhang et al., 2017). Histone acetylation is regulated by histone acetyltransferase (HAT) and histone deacetylase (HDAC). Accumulating evidence has revealed that HDACs may be potential therapeutical targets for the treatment of AD (Xu et al., 2011), especially regarding the involvement of HDAC3 in the molecular mechanisms of AD (Bardai and D’Mello, 2011; McQuown et al., 2011). Interestingly, a study in ankylosing spondylitis found that HDAC3 formed negative feedback loop with miR-130a in peripheral blood mononuclear cells (Jiang and Wang, 2016). Nevertheless, it is not clarified whether inhibiting HDAC3 regulates the expression of miR-130a and the ratio of AQP4-M1/M23 in AD.

Some researchers have recognized the beneficial effects of intermittent fasting (IF) against AD (Stewart et al., 1989; Halagappa et al., 2007). What is more, IF results in ketone body metabolism and an increase in β-hydroxybutyrate (βOHB; a major component of ketone bodies) level in blood (Robinson and Williamson, 1980; Wang et al., 2017). βOHB has been reported to traverse the blood–brain barrier and significantly inhibit HDAC3 expression as an endogenous pan HDACs inhibitor (Shimazu et al., 2013). Emerging evidence has shown that βOHB exerts a protective effect against AD (Tarasoff-Conway et al., 2015; Xie et al., 2015). Therefore, it is needed to demonstrate whether βOHB plays a key role in the effects of IF against AD, which may be associated with the inhibition of HDAC3.

In the present study, we confirmed the beneficial effects of IF in AD, and the mechanism may be associated with recovery of AQP4 polarity, resulting from the reduction of AQP4-M1/M23 ratio. Furthermore, βOHB may at least partly mediate the effect of IF on the reduction of AQP4-M1/M23 ratio in AD, in which miR-130a and HDAC3 are possibly implicated.

## Materials and Methods

### Reagents

β-hydroxybutyrate (βOHB) was purchased from Shanghai Yingxin Laboratory Equipment Co., Ltd. (Shanghai, China); rabbit anti-Aβ_1-42_ polyclonal antibody (ab10148) was purchased from Abcam Inc. (Cambridge, United Kingdom); rabbit anti-HDAC3 polyclonal antibody (sc-11417) and rabbit anti-β-actin polyclonal antibody (sc-130656) were obtained from Santa Cruz Biotechnology (Santa Cruz, CA, United States). Rabbit anti-AQP4 polyclonal antibody (D160223), goat anti-rabbit immunoglobulin G (IgG) secondary antibody, and FITC-donkey anti-rabbit IgG secondary antibody were obtained from Shanghai Sangon Biotech Co., Ltd. (Shanghai, China). Cell culture medium was purchased from Hyclone Laboratories Inc. (United States). Aβ_25-35_ (American Peptide Company, Inc., United States), a toxic fragment of the full-length Aβ peptide, was solubilized in sterile water and aggregated at 37°C for 7 days. Immunohistochemistry kits were obtained from Beijing Zhongshan Biotechnology (Beijing, China).

### *In Vivo* Study

#### Animals and Treatment

APP/PS1 double-transgenic mice [B6C3-Tg (APPswe, PS1dE) 85Dbo/J] and wild-type (WT) littermates were obtained from Jackson Laboratory (United States) and housed in the temperature-controlled 12h-light/12h-dark environment. Impaired spatial learning and brain Aβ deposits are developed in APP/PS1 mice at 6–7 months of age (Jankowsky et al., 2004; Reiserer et al., 2007). Protocols were approved by the Animal Care and Use Committee of China Medical University. In this study, alternate-day fasting (ADF) was used as a means of IF. At 5 months of age, WT mice were grouped into WT and WT+ADF, and APP/PS1 mice were grouped into AD and AD+ADF. Mice in WT and AD groups were fed *ad libitum* and mice in WT+ADF and AD+ADF groups were fed *ad libitum* every other day and fasted the following day. Each group had five males and five females, with roughly balanced body weights across the groups. Body weight was measured once a week. After 5 months, mice were used to perform behavioral tests, and then sacrificed. Their brains were collected, weighed, and divided into halves, the left of which was stored in 4% paraformaldehyde (pH 7.4) for immunohistochemistry and immunofluorescence tests, and the right of which was stored at -80°C and then used for quantitative reverse transcriptase (qRT)-polymerase chain reaction (PCR) and Western blot analyses.

#### Behavior

Morris water maze test was carried out as previously described in detail (Zhang et al., 2017). Briefly, in escape training trials (4 consecutive days), a hidden escape platform (8 cm in diameter) was placed 1 cm under the water surface. Each mouse was tested four trials with an inter-trial interval of 60 s per day. Latency to escape onto the hidden platform was recorded. In the probe trial (day 5), mice swam freely for 60 s in the water tank without a hidden platform. The times of pass through the former platform region, as well the residence time, were separately recorded.

#### Immunohistochemistry

Three coronal sections (5 mm thick between 2.5 and 3.5 mm posterior to bregma, separated serially by 200–300 μm) from each mouse were used for Aβ_1-42_ immunohistochemistry with the same method as previously described (Zhang et al., 2017). In brief, sections were incubated overnight with primary rabbit anti-Aβ_1-42_ polyclonal antibody (1:200) and developed with goat anti-rabbit IgG secondary antibody. Aβ-immunopositive plaques in the whole cerebral cortex of selected sections were observed and counted by blind method under an optical microscope. Results were expressed as the average number of Aβ plaques.

#### Immunofluorescence

Immunofluorescence was performed for AQP4 staining using the same method with immunohistochemistry as described until incubated with primary antibody (Zhang et al., 2017). The sections were incubated with primary rabbit anti-AQP4 polyclonal antibody (1:200) at 4°C overnight, subsequently, incubated in dark with FITC-coupled donkey anti-rabbit secondary antibody (1:50) for 30 min. DAPI was used as a nuclear stain, washed and finally mounted in glycerol containing 1% n-propyl gallate (Sigma Chemical Co.). Sections were observed and photographed in a Fluorescence Microscope (Nikon 80i, Japan). Images were compounded using FV10-ASW 2.1 Viewed software.

#### qRT-PCR Assay of Cortex Samples

Total RNA from thawed cerebral cortex was prepared with SV Total RNA Isolation System kit (Promega Corporation, Madison, WI, United States) and reverse-transcribed with Prime-Script RT-PCR System kit (TaKaRa Dalian Biotechnology, Dalian, China). MicroRNAs were isolated with SanPrep Column microRNA Mini-Preps Kit (Shanghai Sangon Biotech Co., Ltd., China) and reverse-transcribed with microRNAs First Strand cDNA Synthesis (Shanghai Sangon Biotech Co., Ltd., China). The resulting complementary DNAs were used as templates for Real-Time PCR in the ABI 7500 Real-Time PCR system (Applied Biosystems, Inc., Carlsbad, CA, United States) with SYBR Premix Ex Taq Mix (TaKaRa Dalian Biotechnology, Dalian, China) as we have described previously (Zhang et al., 2017). The primer sequences for Real-Time PCR are listed as follows: mus AQP4, forward: 5′-TTAATGAAGTGCATAGTGCCG-3′, reverse: 5′-GCATTGTTTCATGGCTCG-3′ (117 bp product); mus AQP4-M1, forward: 5′-AGGCGGTGGGGTAAGTGT-3′, reverse: 5′-TATGGTGGATCCCACACCGA-3′ (150 bp product); mus AQP4-M23, forward: 5′-GGAAGGCTAGGTTGGTGACTTC-3′, reverse: 5′-TGGTGACTCCCAATCCTCCAAC-3′ (460 bp product); mus HDAC3, forward: 5′-ATCCGCCAGACAATCTTTGA-3′, reverse: 5′-CTCGGGACCTCTCTCTTCAG-3′ (132 bp product); β-actin forward: 5′-CATCCGTAAAGACCTCTATGCCAAC-3′, reverse: 5′-ATGGAGCCACCGATCCACA-3′ (171 bp product); mmu-miR-130a-3p, forward: 5′-GCAGTGCAATGTTAAAAGGGCAT-3′, reverse: 5′-CGCTTCACGAATTTGCGTGTCAT-3′; U6, forward: 5′-GCTTCGGCAGCACATATACTAAAAT-3′, reverse: 5′-CGCTTCACGAATTTGCGTGTCAT-3′. β-actin (constitutive gene) mRNA or U6 was used as an internal control. Data were expressed by comparative *C*_T_ method (also known as the 2^-ΔΔC_T_^ method).

#### Western Blot Analysis of Cortex Samples

Western blot was performed as we have described in detail elsewhere (Zhang et al., 2017). Briefly, thawed cerebral cortex samples were directly homogenized in RIPA buffer containing 0.1% protease inhibitor (Amerso, United States). The protein concentration was determined using the Bradford method with Coomassie Brilliant Blue (CBB G-250). Equal amounts of soluble protein were separated by SDS–PAGE and transferred onto a polyvinylidene fluoride membrane. Immunoblotting was performed with antibodies specific for rabbit anti-AQP4 (1:1000), anti-HDAC3 (1:1000), or anti-β-actin (1:1000). Primary antibodies were visualized using goat anti-rabbit IgG secondary antibody (1:3000) and a chemiluminescent detection system (ECL reagent; Thermo Fisher Scientific Inc.). Variations in sample loading were normalized relative to β-actin.

### *In Vitro* Study

#### Cell Culture

Human U251 glioma cells were obtained from Chinese academy of sciences cell bank (KCB200965YJ, Kunming, China) and cultured at 37°C with 5% CO_2_ in DMEM-high glucose medium with 10% fetal bovine serum, 100 U/ml penicillin, and 100 μg/ml streptomycin. Cells were passaged about twice a week and detached using 0.25% trypsin.

#### qRT-PCR and Western Blot Analyses of Cells

Cells were incubated in 6-well culture microplates in 2 ml antibiotic-free medium, and cultured for 3 h with βOHB treatment at a final concentration of 1 mM (βOHB and βOHB+Aβ groups) or without βOHB treatment (control and Aβ groups). The βOHB concentration was selected based on previous MTS results and the obtainable level of βOHB *in vivo* (Robinson and Williamson, 1980). After 3 h, the cells in Aβ and βOHB+Aβ groups were treated with Aβ_25-35_ (final concentration 2 or 10 μM), and then cultured for an additional 12 h. Subsequently, cells were collected and used for mRNA, microRNA, and protein expression assays by qRT-PCR and western blot as described above. Specifically, levels of miR-130a, AQP4, and HDAC3 mRNA in cells were analyzed. The following primer sequences were used: homo AQP4, forward: 5′-GTGATTCCAAACGGACTGATG-3′, reverse: 5′-TTGGTCTTTCCCCTTCTTCTC-3′ (413 bp product); homo AQP4 M1, forward: 5′-GGCATGAGTGACAGACCCAC-3′, reverse: 5′-TCATACTGAAGACAATACCT-3′ (975 bp product); homo AQP4-M23, forward: 5′-ATCATGGTGGCTTTCAAAGG-3′, reverse: 5′-TCATACTGAAGACAATACCT-3′ (909 bp product); homo HDAC3, forward: 5′-GAGGGATGAACGGGTAGACA-3′, reverse: 5′-CAGGTGTTAGGGAGCCAGAG-3′ (137 bp product); β-actin, forward: 5′-CATCCGTAAAGACCTCTATGCCAAC-3′, reverse: 5′-ATGGAGCCACCGATCCACA-3′ (171 bp product); hsa-miR-130a-3p, forward: 5′-CCAGTGCAATGTTAAAAGGGCAT-3′, reverse: 5′-CGCTTCACGAATTTGCGTGTCAT-3′; U6, forward: 5′-GCTTCGGCAGCACATATACTAAAAT-3′, reverse: 5′-CGCTTCACGAATTTGCGTGTCAT-3′. Levels of proteins were analyzed with the corresponding primary antibodies: anti-AQP4 (1:1000), anti-HDAC3 (1:1000), and anti-β-actin (1:1000) antibody. This experiment was carried out in duplicate and repeated three times.

#### Transfection of MicroRNA Mimic and Inhibitor

The micrOFF^®^ miRNA mimic and inhibitor for human miR-130a were designed and synthesized by Guangzhou RiboBio Co., Ltd. (Guangzhou, China). miR-130a mimic sequence: 5′-CAGUGCAAUGUUAAAAGGGCAU-3′, anti-sequence: 5′-GUCACGUUACAAUUUUCCCGUA-3′; and miR-130a inhibitor sequence: 5′-mAmUmGmCmCmCmUmUmUmUmAmAmCmAmUmUmGmCmAmCmUmG-3′ (mN, 2′-O-methyl ribose). Cells were seeded in 6-well culture microplates in 2 ml antibiotic-free medium, and then incubated with 200 nM miRNA mimic or inhibitor according to the manufacturer’s protocol (ribo FECT^TM^ CP Transfection Kit; Guangzhou RiboBio Co., Ltd.). The transfected cells were incubated at 37°C for 24 h. The micrOFF^®^ miRNA mimic control and micrOFF^®^ miRNA inhibitor control (Guangzhou RiboBio Co., Ltd.) were used as controls, respectively. Subsequently, cells were collected and total mRNA, microRNA, and protein were extracted. The expression of AQP4 mRNA and protein, and miR-130a levels were investigated by the above methods. This experiment was repeated three times and carried out in duplicate.

#### HDAC3 Down-regulation by Small Interfering RNA (siRNA) and HDAC3-Specific Inhibitor

HDAC3 siRNA duplex (Guangzhou RiboBio Co., Ltd.) or RGFP966 (Selleck Chemicals Co., Ltd.) was used to interfere with endogenous HDAC3 mRNA levels. siRNA was performed with siRNA transfection reagent (Guangzhou RiboBio Co., Ltd.) as we have described in detail previously (Zhang et al., 2017). Cells were incubated in 6-well culture microplates at 37°C with antibiotic-free medium containing 10 μM RGFP966. After 24 h, the expression of AQP4 mRNA and protein, and miR-130a levels were investigated by the above methods. Untreated cells and non-specific siRNA (scrambled siRNA; Guangzhou RiboBio Co., Ltd.) were used as controls. This experiment was repeated three times and performed in duplicate.

### Statistical Analyses

Statistical analysis of the data was performed by one-way analyses of variance (ANOVAs) and Fisher’s least significant difference (LSD) multiple comparison *post hoc* tests in SPSS 13.0 software for Windows (version 13.0; SPSS, Chicago, IL, United States). Morris water maze escape latency data were analyzed with two-way repeated measures ANOVA. Data are presented graphically as means ± standard deviations (SDs). Probability values (*p*-values) less than 0.05 (*p* < 0.05) were considered statistically significant.

## Results

### Sign, Body Weight, and Brain–Body Weight Ratio

During the treatment period, there were no abnormal signs in the mice, except that one female mouse in AD group died at 9-month old. No significant difference was found in body weight or brain–body weight ratio among groups (data not reported).

### ADF Ameliorated the Cognitive Dysfunction of APP/PS1 Mice

**FIGURE 1:**
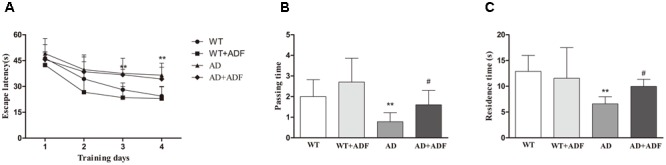
Assay of mice behavior in Morris water maze. Morris water maze test was conducted to analyze spatial learning and memory in a circular pool filled with milk-opacified water. The procedure consisted of escape trials (4 consecutive days) and a probe trial (day 5). In escape trials, a hidden escape platform, 8 cm in diameter, was placed 1 cm under the water surface. Each mouse was tested 4 trials with an inter-trial interval of 60 s per day. In the probe trial, mice swam freely for 60 s in the water tank after the platform was removed. **(A)** Latency to escape onto the hidden platform in escape trials. **(B)** The number of pass through the former escape platform area (passing time) in the probe trial. **(C)** The residence time in the region of the former platform location (residence time) in the probe trial (*n* = 9 or 10; mean ± SD; two-way ANOVA with repeated measures for escape latency and one-way ANOVA for passing time both followed by LSD multiple comparison tests; ^∗∗^*p* < 0.01 vs. WT group, ^#^*p* < 0.05 vs. AD group).

Cognitive dysfunction is relevant to the clinical symptomatology of AD. Morris water maze was conducted to examine spatial learning and memory. In escape training trials (**Figure 1A**), the escape latencies were significantly longer (*p* < 0.01) in APP/PS1 mice on 3–4 days than those in WT mice. With ADF intervention, there was a trend but without statistical significance (*p* > 0.05) in alleviating the extended escape latencies for APP/PS1 mice on 3–4 days. In the probe trial (**Figures 1B,C**), a significant reduction (*p* < 0.01) in passing time or residence time was found in APP/PS1 mice compared with WT mice. However, passing time or residence time was remarkable increased (*p* < 0.05) in ADF-treated APP/PS1 mice relative to APP/PS1 mice.

### ADF Protected against the Increase of Aβ Plaques in the Cerebral Cortex of APP/PS1 Mice

**FIGURE 2:**
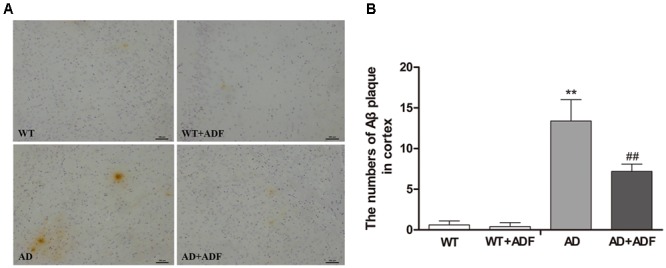
Alternate-day fasting (ADF) protected against the increase of Aβ plaques in the cerebral cortex of APP/PS1 mice. **(A)** Brown immunoreactive plaques indicate the Aβ deposition in the cerebral cortex of mice (bars = 100 μm). **(B)** The average number of Aβ plaques in the whole cerebral cortex from each section (*n* = 9 or 10; mean ± SD; one-way ANOVA followed by LSD multiple comparison tests; ^∗∗^*p* < 0.01 vs. WT group, ^##^*p* < 0.05 vs. AD group; magnify 100×).

To explore whether ADF alleviates the increase of Aβ deposition, immunohistochemistry was used to examine the number of Aβ plaques in the cerebral cortex (**Figure 2**). Brown plaques indicate the localization of Aβ immunoreactivity in mice brains. Compared with WT mice, a marked increase (*p* < 0.01) in the number of Aβ immunopositive plaques was found in the cerebral cortex of APP/PS1 mice; however, there was a significant decrease (*p* < 0.01) in ADF-treated APP/PS1 mice compared with APP/PS1 mice.

### ADF Alleviated the Loss of AQP4 Polarity in the Cerebral Cortex of APP/PS1 Mice

**FIGURE 3:**
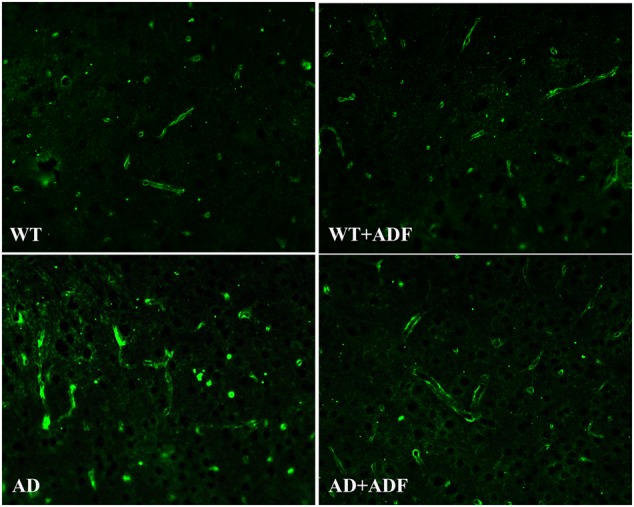
Alternate-day fasting alleviated the loss of AQP4 polarity in the cerebral cortex of APP/PS1 mice. Immunofluorescence staining for AQP4 showed stronger intensity on the astrocytic endfeet in the brains of WT mice with or without ADF treatment. In the brains of APP/PS1 mice, immunofluorescence staining for AQP4 showed AQP4 localization was severely perturbed, exhibiting weaker intensity on the astrocytic endfeet and stronger intensity on the somal. After the ADF intervention, the polarity of AQP4 recovered in the APP/PS1 mice brains.

To investigate whether ADF alleviates the loss of AQP4 polarity, immunofluorescence was performed for AQP4 staining to observe the polarity changes (**Figure 3**). Immunoflorescent labeling demonstrated that AQP4 expression was highly localized to astrocytic endfeet, showing a polar distribution, in the cerebral cortex of WT mice with or without ADF treatment. In the cerebral cortex of APP/PS1 mice, AQP4 localization was severely perturbed, exhibiting a loss of polarity to the astrocytic endfeet and an increase of somal labeling. After ADF intervention, the polarity of AQP4 was recovered in APP/PS1 mice brains.

### ADF Alleviated the Increase of AQP4-M1/M23 Ratio in the Cerebral Cortex of APP/PS1 Mice

**FIGURE 4:**
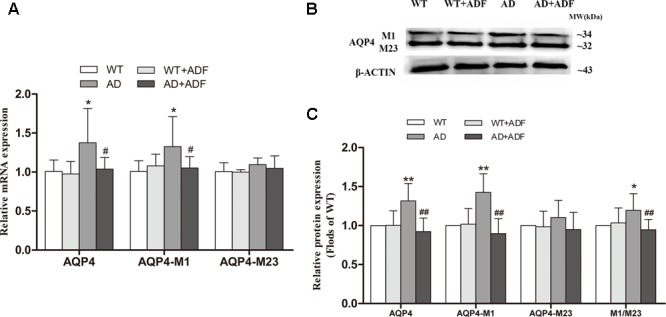
Alternate-day fasting alleviated the increase of AQP4-M1/M23 ratio in the cerebral cortex of APP/PS1 mice. The relative expression of AQP4 mRNA (β-actin as a reference standard) **(A)** and proteins **(B,C)** were analyzed by qRT-PCR and Western blot, respectively (*n* = 9 or 10; mean ± SD; one-way ANOVA followed by LSD multiple comparison tests; ^∗^*p* < 0.05, ^∗∗^*p* < 0.01 vs. WT group, ^#^*p* < 0.05, ^##^*p* < 0.01 vs. AD group).

To explore the possible mechanisms on the role of ADF in AQP4 polarity, we examined the ratio of AQP4-M1/M23 in the cerebral cortex (**Figure 4**). Compared with WT mice with or without ADF treatment, the expression of AQP4 and AQP4-M1 mRNA and protein were increased (*p* < 0.05; *p* < 0.01) in the cerebral cortex of APP/PS1 mice. Meanwhile, APP/PS1 mice with ADF treatment had a significant decrease (*p* < 0.05; *p* < 0.01) in AQP4 and AQP4-M1 mRNA and protein expression in the cerebral cortex relative to APP/PS1 mice. However, no differences in AQP4-M23 mRNA and protein expression were found in the cerebral cortex among groups. In addition, the increase of AQP4-M1/M23 protein ratio was also observed in APP/PS1 mice, which were reversed by ADF (*p* < 0.05; *p* < 0.01).

### ADF Alleviated the Decrease of miR-130a Expression and the Increase of HDAC3 Expression in the Cerebral Cortex of APP/PS1 Mice

**FIGURE 5:**
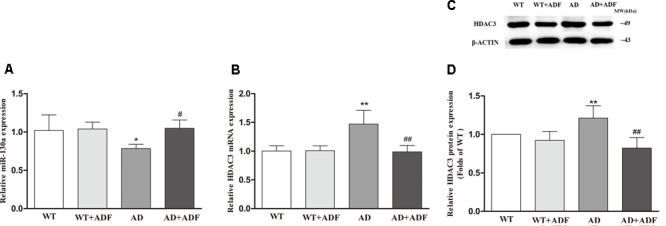
Alternate-day fasting alleviated the decrease of miR-130a expression and the increase of HDAC3 expression in the cerebral cortex of APP/PS1 mice. The relative expression of miR-130a (**A**; U6 as a reference standard), HDAC3 mRNA (**B**; β-actin as a reference standard), and proteins **(C,D)** were analyzed by qRT-PCR and Western blot, respectively (*n* = 9 or 10; mean ± SD; one-way ANOVA followed by LSD multiple comparison tests; ^∗^*p* < 0.05, ^∗∗^*p* < 0.01 vs. WT group, ^#^*p* < 0.05, ^##^*p* < 0.01 vs. AD group).

To further investigate the possible mechanisms responsible for the role of ADF in AQP4-M1/M23 ratio, we examined the expression of miR-130a (**Figure 5A**) and HDAC3 (**Figures 5B–D**) in the cerebral cortex among groups. The results showed that the expression of miR-130a was significantly decreased (*p* < 0.05) and the expression of HDAC3 mRNA and protein was significantly increased (*p* < 0.01) in APP/PS1 mice compared with WT mice. After ADF intervention, a marked increase (*p* < 0.05) in miR-130a expression and a significant decrease (*p* < 0.01) in HDAC3 expression were found in APP/PS1 mice.

### βOHB Alleviated the Increase of AQP4-M1/M23 Ratio in Aβ-Exposed U251 Cells

**FIGURE 6:**
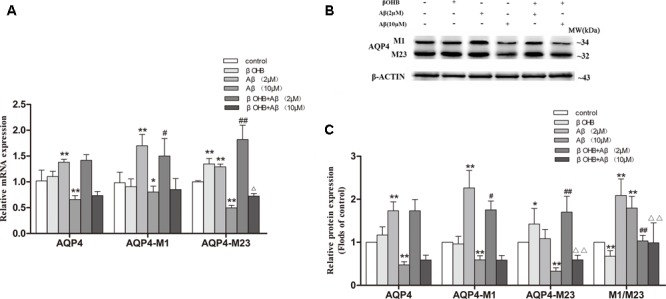
β-Hydroxybutyrate (βOHB) alleviated the increase of AQP4-M1/M23 ratio in Aβ-exposed U251 cells. The relative expression of AQP4 mRNA (β-actin as a reference standard) **(A)** and proteins **(B,C)** were analyzed by qRT-PCR and Western blot, respectively [*n* = 6; mean ± SD; one-way ANOVA followed by LSD multiple comparison tests; ^∗^*p* < 0.05, ^∗∗^*p* < 0.01 vs. control group, ^#^*p* < 0.05, ^##^*p* < 0.01 vs. Aβ (2 μM) group, ∆*p* < 0.05, ∆∆*p* < 0.01 vs. Aβ (10 μM) group].

It is known that ADF results in an increase of endogenous βOHB, and our preliminary experiments also showed that the level of βOHB began to rise significantly after fasting for 12 h. To investigate whether βOHB plays a role in regulating AQP4-M1/M23 ratio by ADF, we detected the ratio of AQP4-M1/M23 *in vitro* (**Figure 6**). Cells exposed to 2 μM Aβ had a significant increase in AQP4 mRNA and protein expression compared with those observed in control cells (*p* < 0.05; *p* < 0.01). However, AQP4 mRNA and protein expression was obviously decreased (*p* < 0.05; *p* < 0.01) in cells exposed to 10 μM Aβ. Meanwhile, an increase in AQP4-M23 expression was observed in βOHB-pre-treated cells compared with control cells (*p* < 0.05), as well in Aβ-exposed cells with βOHB pre-treatment compared with cells only treated with Aβ (*p* < 0.01). A decrease in AQP4-M1 expression was observed in βOHB-pre-treated cells exposed to Aβ (2 μM) compared with Aβ-exposed cells (*p* < 0.05). Furthermore, we also found that the AQP4-M1/M23 ratio was remarkably increased in cells exposed to 2 or 10 μM Aβ, which was ameliorated by βOHB pre-treatment (*p* < 0.01). The protein band was also stained by Red ponceau (see Supplementary Figure S1).

### βOHB Alleviated the Decrease of miR-130a Expression and the Increase of HDAC3 Expression in Aβ-Exposed U251 Cells

**FIGURE 7:**
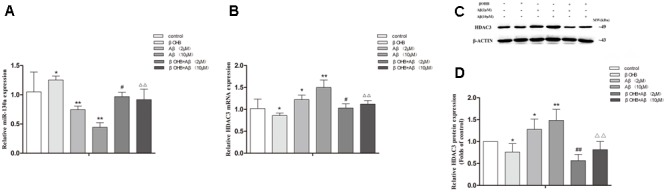
β-Hydroxybutyrate alleviated the decrease of miR-130a expression and the increase of HDAC3 expression in Aβ-exposed U251 cells. The relative expression of miR-130a (**A**; U6 as a reference standard), HDAC3 mRNA (**B**; β-actin as a reference standard), and proteins **(C,D)** were analyzed by qRT-PCR and Western blot, respectively [*n* = 6; mean ± SD; one-way ANOVA followed by LSD multiple comparison tests; ^∗^*p* < 0.05, ^∗∗^*p* < 0.01 vs. control group, ^#^*p* < 0.05, ^##^*p* < 0.01 vs. Aβ (2 μM) group, ∆∆*p* < 0.01 vs. Aβ (10 μM) group].

Cells exposed to Aβ had a significant decrease in miR-130a expression (**Figure 7A**) and a remarkable increase in HDAC3 expression (**Figures 7B–D**) relative to those observed in control cells (*p* < 0.05; *p* < 0.01). The increase of miR-130a expression and the decrease of HDAC3 expression were observed in βOHB-pre-treated cells compared with control cells (*p* < 0.05). Meanwhile, βOHB-pre-treated cells exposed to Aβ had a significant increase in miR-130a expression and an obvious decrease in HDAC3 expression relative to the levels observed in Aβ-exposed cells (*p* < 0.05; *p* < 0.01).

### Effect of miR-130a on Transcriptional Activity of AQP4 and the Ratio of AQP4-M1/M23

**FIGURE 8:**
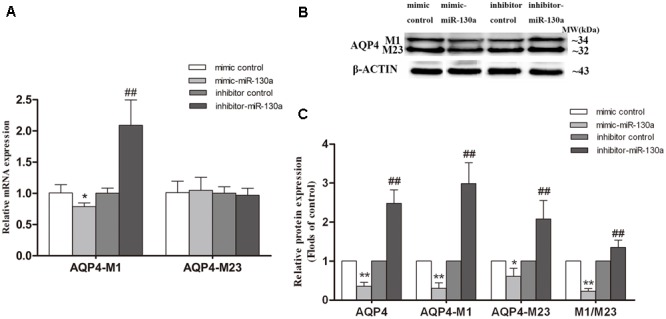
Effect of miR-130a on transcriptional activity of AQP4 and the AQP4-M1/M23 ratio. The miR-130a mimic and inhibitor were separately used to improve and inhibit miR-130a expression. The relative expression of AQP4 mRNA **(A)** and proteins **(B,C)** were analyzed by qRT-PCR (U6 as a reference standard) and Western blot, respectively, in miR-130a mimic and inhibitor-treated cells (*n* = 6; mean ± SD; one-way ANOVA followed by LSD multiple comparison tests; ^∗^*p* < 0.05, ^∗∗^*p* < 0.01 vs. mimic control group, ^##^*p* < 0.01 vs. inhibitor control group).

To further investigate whether miR-130a alters the AQP4-M1/M23 ratio through regulating the transcriptional activity of AQP4-M1, miR-130a mimic and inhibitor were used to explore the possible mechanism in U251 cells (**Figure 8**). We found that the expression of AQP4-M1 mRNA was decreased in cells treated with miR-130a mimic (*p* < 0.05) and was increased in cells treated with miR-130a inhibitor (*p* < 0.01). However, no difference in AQP4-M23 mRNA expression was found in cells treated with miR-130a mimic or inhibitor. Moreover, the expression of AQP4-M1 and AQP4-M23 protein was decreased in different degrees (*p* < 0.05; *p* < 0.01), resulting in the decrease of AQP4-M1/M23 protein ratio (*p* < 0.01), in cells treated with miR-130a mimic, and increased in varying extent in AQP4-M1 and AQP4-M23 protein expression, leading to the increase of AQP4-M1/M23 protein ratio in cells treated with miR-130a inhibitor (*p* < 0.01). The protein band was also stained by Red ponceau (see Supplementary Figure S2).

### Silencing HDAC3 Caused the Up-regulation of miR-130a Expression and the Reduction of AQP4-M1/M23 Ratio in U251 Cells

**FIGURE 9:**
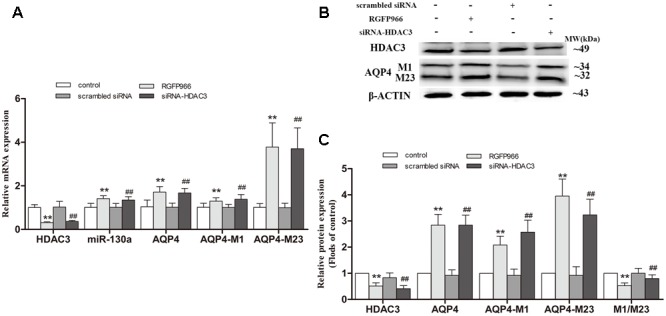
Silencing HDAC3 caused the up-regulation of miR-130a expression and reduction of AQP4-M1/M23 ratio in U251 cells. siRNA duplex or RGFP966 was used to interfere with endogenous HDAC3 expression (untreated cells and non-specific siRNA as controls). The relative expression of miR-130a (**A**; U6 as a reference standard), AQP4 mRNA (**A**; β-actin as a reference standard), and proteins **(B,C)** expression were, respectively, analyzed by qRT-PCR and Western blot in HDAC3-silenced cells (*n* = 6; mean ± SD; one-way ANOVA followed by LSD multiple comparison tests; ^∗∗^*p* < 0.01 vs. control group, ^##^*p* < 0.01 vs. scrambled siRNA group).

To further determinate whether HDAC3 regulates miR-130a expression, siRNA duplex or specific inhibitor RGFP966 was used to interfere with endogenous HDAC3 mRNA expression in cells (**Figure 9**). As illustrated in **Figure 9A**, the expression of miR-130a was significantly increased in cells with HDAC3 siRNA or RGFP966 (*p* < 0.01). Interestingly, in HDAC3-silenced cells, we found the expression of AQP4-M1 and AQP4-M23 mRNA and protein were increased in different degrees (*p* < 0.01), however, the AQP4-M1/M23 protein ratio was decreased compared with the levels in controls (*p* < 0.01). The protein band was also stained by Red ponceau (see Supplementary Figure S3).

## Discussion

The pathogenesis of AD is complex, involving multiple mechanisms, which causes considerable difficulty in developing an effective treatment. Pharmacologic therapies approved for AD currently are mainly several acetylcholinesterase inhibitors (donepezil, rivastigmine, and galantamine) and the *N*-methyl-D-aspartate receptor antagonist (memantine). However, these drugs focus on delaying or arresting disease progression rather than reversing disease. The importance of IF in general in forestalling the onset of certain neurodegenerative disorders, including AD, has been documented in numerous studies (Stewart et al., 1989; Halagappa et al., 2007). Results from Morris water maze in this study showed that the impairment of spatial memory was ameliorated by ADF in APP/PS1 mice. ADF was also found to play a role in protecting against the increase of Aβ deposition in the brains of APP/PS1 mice. As is well-known, ADF results in an increase in the serum level of the ketone body (Robinson and Williamson, 1980; Wang et al., 2017). The increased ketone body by fasting or ketogenic diet is remarkably effective in managing intractable seizures in children. Moreover, βOHB accounts for about 70% of ketone body. Accumulation of βOHB in blood increases to 1–2 mM during fasting when the liver switches to fatty acid oxidation (Shimazu et al., 2013). In our preliminary experiments, we found the level of βOHB in blood was increased after fasting 12 h in C57BL/6 mice. Notably, βOHB has been reported to take effect in delaying AD progression in several studies (Findlay et al., 2015; Xie et al., 2015). It is suggested that βOHB may play a crucial role in protective function of ADF against AD.

Progressive accumulation and aggregation of Aβ in the brain are thought to be a net result of imbalance between their production and clearance (Zlokovic et al., 2000); indeed, Aβ clearance seems to be impaired in both early and late forms of AD (Mawuenyega et al., 2010). AQP4 has been indicated to be closely related with Aβ clearance (Iliff et al., 2012). In some studies, no differences were found in the expression of AQP4 in the frontal cortex between AD patients and controls (Rodriguez et al., 2006; Perez et al., 2007), while one study reported that the expression of AQP4 was significantly decreased in stage IV–V AD patients with moderate or severe cerebral amyloid angiopathy (Moftakhar et al., 2010). However, the present study showed a significant increase in the expression of AQP4 in the brains of APP/PS1 mice, which is in accordance with some human and animal studies (Hoshi et al., 2012; Xu et al., 2015).The above inconsistent results in AQP4 expression in AD may be associated with the different stages of disease, specific neuroanatomical areas, and whether patients had complications or not. In addition, several animal models of AD studies have shown a redistribution of astrocytic AQP4 from endfeet membranes to non-endfeet ones, resulting in a loss of AQP4 polarity (Yang et al., 2011; Xu et al., 2015). Immunofluorescence results from this study also demonstrated that AQP4 localization was severely perturbed, exhibiting a loss of polarity to the endfeet and increased somal labeling. It is indicated that although the expression of AQP4 was increased in APP/PS1 mice, the role of AQP4 in Aβ clearance failed to play due to the loss of AQP4 polarity. Furthermore, Deng et al. (2014) found that the ratio of AQP4-M1/M23 was increased in the brain after hypoglycemia which may result in the loss of AQP4 polarity. In this study, the expression of AQP4-M1 was increased but no significant alteration was found in AQP4-M23 expression in the cerebral cortex of APP/PS1 mice, consequently leading to a marked increase in AQP4-M1/M23 ratio. Meanwhile, this study showed that ADF alleviated the loss of AQP4 polarity, up-regulation of AQP4-M1 expression, and increase of AQP4-M1/M23 ratio in APP/PS1 mice. It is possible that ADF inhibited the increase of AQP4-M1/M23 ratio in AD through down-regulating the expression of AQP4-M1, accordingly recovered AQP4 polarity. However, further study is needed to explore whether other pathways exist to affect AQP4 polarity in AD by ADF. Insulin-degrading enzyme (IDE) is involved in Aβ clearance and associated with the ketone body, thus it is also necessary to determine whether ADF reduces Aβ deposition through regulating IDE or other enzymes involved in the degradation of Aβ. Other mechanisms on Aβ clearance are equally worth studying in the effect of ADF on AD.

The results from *in vitro* study indicated that the expression of AQP4 was increased with exposure to low concentrations of Aβ (2 μM) and decreased with exposure to high concentrations of Aβ (10 μM) in U251 cells, which is consistent with the results of Yang et al. (2012) studies. Low concentration of Aβ possible promotes astrocyte activation, causing the up-regulation of AQP4 as a compensatory response, while high concentration of Aβ likely results in astrocyte dysfunction and lowering the expression of AQP4. In this study, the expression of AQP4 was shown to be increased in APP/PS1 mice, which is in agreement with results of low concentration of Aβ *in vitro*. Further study is needed to explore whether the expression of AQP4 is decreased in AD mice at an older age with an increase in Aβ. More importantly, we found that AQP4-M1/M23 ratio was increased in U251 cells treated with Aβ at both low and high concentration, suggesting that the change of AQP4-M1/M23 ratio may play a more vital role in AD. Furthermore, βOHB was found to alleviate the increase of AQP4-M1/M23 ratio in Aβ-exposed U251 cells. Also, the effect of βOHB on AQP4-M1 was in accordance with that of ADF. Unlike ADF, βOHB was found to increase the expression of AQP4-M23. It is indicated that other ways except βOHB may be involved in the regulation of ADF on AQP4-M23 expression. In a word, βOHB may at least partly mediate the effect of ADF on the reduction of AQP4-M1/M23 ratio in AD.

MicroRNAs are small non-coding RNAs capable of regulating gene expression. Although microRNAs are known to mediate translational silencing in the cytoplasm, recent evidence has suggested that some microRNAs may also regulate gene expression at the transcriptional level (Kim et al., 2008; Younger and Corey, 2011). miR-130a has been reported to repress transcriptional activity of AQP4-M1 but not AQP4-M23 in the study of cerebral ischemia (Sepramaniam et al., 2012). In the current study, decreased miR-130a levels were found in the cerebral cortex of APP/PS1 mice and in Aβ-exposed U251 cells, which were alleviated by ADF and βOHB, respectively. Further research, using miR-130a mimic, showed a decrease in the expression of AQP4-M1 instead of AQP4-M23 mRNA *in vitro*. However, the expression of both AQP4-M1 and AQP4-M23 proteins was decreased in cells treated with mimic, what is more, the ratio of AQP4-M1/M23 was decreased. Additionally, the opposite effects on the regulation of AQP4-M1/M23 ratio were obtained by using miR-130a inhibitor and miR-130a mimic. In rat astrocytes primary cultures, a large proportion of AQP4-M23 protein has been reported to derive from AQP4-M1 mRNA translation, which could explain the inconsistent of alteration in AQP4-M23 mRNA and protein expression in cells treated by miR-130a mimic or miR-130a inhibitor. Furthermore, there was a big discrepancy between the effect of mimic-miR-130a on the expression of AQP4-M1 mRNA and protein, suggesting that this mimic-miR-130a possibly influenced translation of the AQP4-M1 mRNA, rather than only transcription of the AQP4-M1 DNA coding sequence. In this study, the effect of miR-130a mimic on AQP4-M1 was in accordance with that of βOHB, but the opposite effects were found in the alteration of AQP4-M23 expression in cells treated with mimic and βOHB. It is indicated that miR-130a partly mediated the effect of βOHB on the AQP4-M1/M23 ratio, in which other ways may be involved.

HDAC3 knockdown or HDAC3 inhibition promoted the expression of miRNA-130a, and HDAC3 was recruited to the promoter region of the gene encoding miR-130a in peripheral blood mononuclear cells (Jiang and Wang, 2016). In this study, ADF and βOHB were found to alleviate the increase of HDAC3 expression in the cerebral cortex of APP/PS1 mice and in Aβ-exposed U251 cells, respectively. Also, the expression of miR-130a was elevated in HDAC3-silenced or HDAC3 inhibitor-treated U251 cells, suggesting that βOHB increased the expression of miR-130a via inhibiting HDAC3 expression, at least in part. Unexpectedly, silencing of HDAC3 gene expression caused up-regulation of AQP4-M1 and AQP4-M23 in different degrees, suggesting that HDAC3 may regulate AQP4 gene expression by completely different mechanisms independently of miR-130a. Therefore, further study is needed to reveal the mechanism concerning the regulation of AQP4 expression by HDAC3. Most importantly, AQP4-M1/M23 ratio was significantly decreased in HDAC3-silenced cells. Possibly, the repression of AQP4-M1 expression by miR-130a contributed to the reduction of AQP4-M1/M23 ratio. In addition, the effect of silencing HDAC3 on AQP4-M23 but not AQP4-M1 is in consistent with that of βOHB, suggesting the effect of βOHB on AQP4-M23 may be partly mediated by HDAC3. Briefly, HDAC3 may be involved in the reduction of AQP4-M1/M23 ratio induced by βOHB, and in which miR-130a may be implicated. Further study is necessary to investigate whether other βOHB-modulated HDAC subtypes are involved in the regulation of AQP4 in AD.

## Conclusion

In the present study, ADF was demonstrated to improve cognitive function and provide protection against Aβ deposition in APP/PS1 mice. The potential anti-Aβ mechanism of ADF may be associated with alleviating the loss of AQP4 polarity by inhibiting the up-regulation of AQP4-M1/M23 ratio. In addition, βOHB may partly mediate the effect of ADF on the reduction of AQP4-M1/M23 ratio in AD, in which the regulation of βOHB on miR-130a and HDAC3 may be implicated as a potential mechanism. Due to the limited sample, the brain Aβ levels were detected only by immunohistochemistry in this study, which should be confirmed by more quantitative assays, i.e., ELISA. In this study, no effects of ADF on WT mice were found, of which the mechanism needs to be studied in the future. In our daily life, IF is easy to implement, which may be utilized as a potential candidate for AD prevention. However, it should be prudent to extrapolate the conclusions from experimental AD models to humans.

## Ethics Statement

The animal experiment was approved by the Animal Care and Use Committee of China Medical University, which complies with the National Institute of Health Guide for the Care and Use of Laboratory Animals.

## Author Contributions

LA conceived and designed the experiments. JZ and ZZ performed the experiments. XL analyzed the data. AX, CJ, and YC contributed reagents and materials. JZ, WS, and LA drafted the manuscript. All authors approved the final version to be published.

## Conflict of Interest Statement

The authors declare that the research was conducted in the absence of any commercial or financial relationships that could be construed as a potential conflict of interest.
