# Supplementary material for: Intermittent Fasting Protects against Alzheimer’s Disease Possible through Restoring Aquaporin-4 Polarity
Source: Front Mol Neurosci. 2017 Nov 29;10:395. doi: 10.3389/fnmol.2017.00395 (PMC5712566; doi:10.3389/fnmol.2017.00395)
Supplement: Supplementary file 1 [file Image_1.pdf]

S1. The following figure is a supplement material to Figure 6. The protein band was immunostained by AQP4 antibody and stained by Red ponceau, respectively.

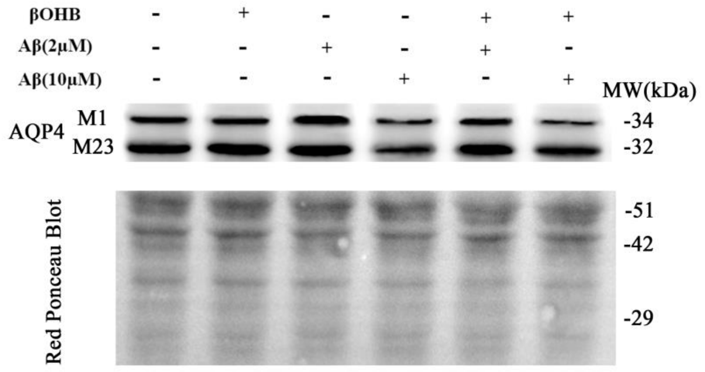

Figure S1.βOHB ameliorated the increase of AQP4-M1/M23 ratio in Aβ-treated U251 cells. (n=6)

S2. The following figure is a supplement material to Figure 8. The protein band was immunostained by AQP4 antibody and stained by Red ponceau, respectively.

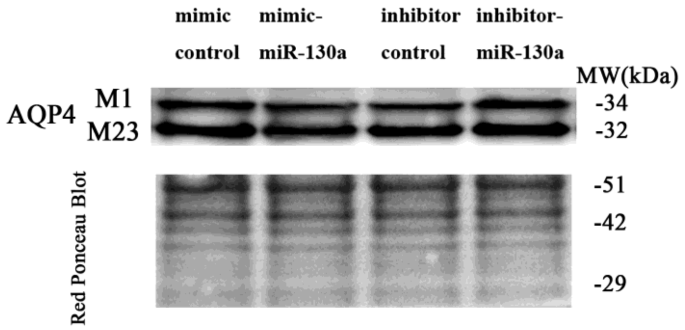

Figure S2.Effect of miR-130a on transcriptional activity of AQP4 and the AQP4-M1/M23 ratio. (n=6)

S3. The following figure is a supplement material to Figure 9. The protein band was immunostained by AQP4 antibody and stained by Red ponceau, respectively.

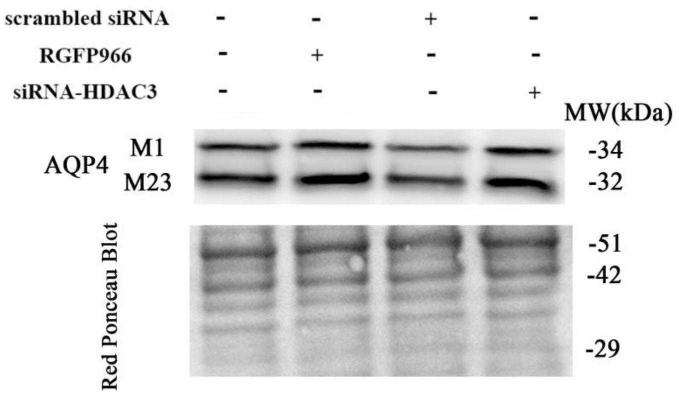

Figure S3. Silencing HDAC3 caused the reduction of AQP4-M1/M23 ratio in U251 cells. (n=6)
